# Mortality due to breast cancer in a region of high socioeconomic vulnerability in Brazil: Analysis of the effect of age-period and cohort

**DOI:** 10.1371/journal.pone.0255935

**Published:** 2021-08-13

**Authors:** Juliana Dantas de Araújo Santos Camargo, Juliano dos Santos, Taynãna César Simões, Jovanka Bittencourt Leite de Carvalho, Glauber Weder dos Santos Silva, Eder Samuel Oliveira Dantas, Weverton Thiago da Silva Rodrigues, Flávio Henrique Miranda de Araújo Freire, Karina Cardoso Meira

**Affiliations:** 1 Graduate Program in Demography at the Federal University of Rio Grande do Norte, Natal, Brazil; 2 Cancer Hospital III, National Cancer Institute, Rio de Janeiro, Rio de Janeiro, Brazil; 3 René Rachou Institute, Oswaldo Cruz Foundation, Belo Horizonte, Minas Gerais, Brazil; 4 Health School, Federal University of Rio Grande do Norte, Natal, Rio Grande do Norte, Brazil; 5 Department of Nursing, Federal University of Rio Grande do Norte, Natal, Rio Grande do Norte, Brazil; 6 Onofre Lopes University Hospital of the Federal University of Rio Grande do Norte, Natal, Brazil; Chung Shan Medical University, TAIWAN

## Abstract

**Introduction:**

Breast cancer is an important public health problem worldwide, with important disparities in incidence, mortality, and survival rates between developed and developing countries due to inequalities regarding access to measures for the prevention and treatment of the disease. In Brazil, there are higher rates of incidence and a downward trend in mortality in regions of greater socioeconomic development.

**Objective:**

To evaluate the effect of age, period, and birth cohort on breast cancer mortality in women aged 20 years and older in the states of the Northeast Region of Brazil, an area of high socioeconomic vulnerability, from 1980 to 2019.

**Methods:**

The death records were extracted from the DATASUS Mortality Information System website (Department of National Health Informatics) from the Ministry of Health of Brazil. Estimable functions were used to estimate the age-period and cohort models (APC) using the Epi library from the R statistical software version 6.4.1.

**Results:**

The average breast cancer mortality rate for the period was 20.45 deaths per 100,000 women. The highest coefficients per 100,000 women were observed in the states of Pernambuco (21.09 deaths) and Ceará (20.85 deaths), and the lowest in Maranhão (13.58 deaths) and Piauí (15.43 deaths). In all of the locations, there was a progressive increase in mortality rates in individuals over 40 years of age, with higher rates in the last five-year period (2015–2019). There was an increase in the risk of death for the five-year period of the 2000s in relation to the reference period (1995–1999) in the Northeast region and in the states of Alagoas, Bahia, Maranhão, Paraíba, and Piauí. In addition, there was an increased risk of death for women born after the 1950s in all locations.

**Conclusion:**

The highest mortality rates in all five-year periods analyzed were observed in states with greater socioeconomic development, with an increase in mortality rates in the 2000s, and a higher risk of death in the younger cohorts.

## Introduction

Breast cancer is the cancer type with the highest incidence and mortality among women, representing 11.6% of new cancer cases and 6.6% of cancer deaths worldwide in 2018 [1]. This reality can be correlated with population aging and changes in women’s reproductive behavior, in addition to changes in eating habits, increased alcohol consumption, and the increased prevalence of overweight and obesity among women [2–5]. Among these risk factors, those with the highest population attributable risk (PAR) are changes in reproductive behavior such as nulliparity, first pregnancy after 30 years of age, increased prevalence of oral contraceptive use, and hormone replacement therapy [2–6].

There are disparities in the temporal evolution of the incidence and mortality of this disease between different regions of the world. This is characterized by a higher incidence in countries with high socioeconomic development, and the highest mortality rates in developing countries. In 2018, the incidence in developed countries was 1.73 times higher than that observed in developing countries (54.4 new cases vs 31.3 new cases per 100 thousand women). The opposite was found in relation to mortality (14.9 deaths vs 11.6 deaths per 100 thousand women) [1].

This reality may be related to the transition from cancer in which a higher incidence of cancers associated with population aging and westernization of habits and lifestyle is expected in locations with a higher socioeconomic development index (SDI), and higher cancer incidence rates associated with infection in locations with less socioeconomic development. The differences in the evolution and magnitude of mortality are related to the determinants of access to early detection, timely treatment, and access to therapeutic innovations [7–10].

Brazil has approximately 270 million inhabitants distributed throughout 26 states and one Federal District. The population is grouped into five geographic regions where the South, Southeast, and Midwest regions present greater socioeconomic development, and the North and Northeast regions greater socioeconomic and health vulnerability. Thus, despite the Brazilian Unified Health System (*Sistema Único de Saúde—*SUS) having universal and free access, disparities regarding access to health persist in the national territory. With regard to Oncology Care, there is greater coverage of mammography, oncology care network, mammography devices, and radiotherapy devices in the most developed regions of the country, contributing to the differences observed in the temporal evolution of breast cancer mortality in Brazil. Due to this, the states and capitals of the most developed regions show a downward temporal evolution, while an inverse pattern is evident in the states and capitals of the Northeast region [11–15].

The temporal evolution of mortality and disease incidence can be dismember into three effects, age, period and cohort. The age effects (A) are related to physiological factors which cause changes in the individual according to age, thus, an increase in the incidence and mortality of chronic non-communicable diseases in older people is expected, as these diseases are associated with long-term exposure to risk factors. The period effects (P) are considered events that occur at specific moments in time, simultaneously influencing all age groups such as world wars, economic expansion or crisis, pandemic and epidemic public health policies, therapeutic innovations, and the expansion of access to health services. The cohort effects (C) are related to factors that affect an entire generation; therefore, they have similar habits and behaviors. Cohort effects are correlated with lifelong exposures which cause changes of different magnitudes in successive age groups and time intervals, and are thus understood as resulting from an interaction between the effects of age and period [16–18].

The evaluation of the effect of temporal events (APC) allows us to raise hypotheses about the temporal evolution of mortality, and the incidence of diseases and health problems, possibly related to changes in the level of population exposure to risk or protection factors (cohort effects) or to changes in diagnostic methods, proposed treatments, access to health services, and improvement in death certificates (period effects). For the breast cancer mortality trend, it is believed that there may be disparities in the period and cohort effects in the Northeast states since the demographic transition, generational changes in the reproductive behavior of women, access to oncology health services, and sexual and reproductive health services, changes in eating habits, alcohol consumption, increased prevalence of overweight and obesity, occurred differently in these locations, with a certain discontinuity in time [16–18].

In view of the importance of breast cancer in the disease burden and mortality for states in the Northeast region, this study aims to analyze the effect of age, period, and cohort (APC) on mortality from breast cancer in the Northeast states of Brazil during the period of 1980 to 2019.

## Materials and methods

### Study design, population and characteristics of the regions

This is an ecological study of a temporal trend that evaluated the effect of age, period, and cohort (APC) on mortality from breast cancer in the North-eastern (NE) states of Brazil from 1980 to 2019. The NE occupies an area of 1,554,291.6 km², covering nine federal units: Alagoas, Bahia, Ceará, Maranhão, Paraíba, Pernambuco, Piauí, Rio Grande do Norte, and Sergipe.

The North-eastern states of Brazil constitute 27.48% of the Brazilian population, and individuals from these states have lower life expectancies at birth as compared to the national one. They also present the same profiles in relation to the values of the Human Development Index (HDI) and per capita household income, with the states of Alagoas, Maranhão, and Piauí presenting the lowest values for these indicators (Table 1). These states also have the highest fertility and unemployment rates in Brazil. The highest fertility rates are observed in Alagoas, Paraíba, and Maranhão, and the highest unemployment rates in the states of Alagoas, Bahia, and Pernambuco (Table 1), showing the socio-economic vulnerability of the North-eastern Brazilian states, and the less aged age structure of the states of Alagoas, Piauí, and Maranhão in relation to the rest of Brazil and the other North-eastern states.

**Table 1:** Sociodemographic characteristics of the states in the Northeast region of Brazil.

**Location**	Population size in 2019 (%)*	Life expectancy at birth **	Fertility rate **
Alagoas	3405893 (1.62)	72.70	1.90
Bahia	15467527 (7.34)	74.20	1.71
Ceará	9128090 (4.33)	74.50	1.73
Maranhão	7083578 (3.36)	71.40	2.12
Paraíba	4074755 (1.93)	74.10	1.76
Pernambuco	9593588 (4.55)	75.00	1.74
Piauí	3229651 (1.53)	71.60	1.74
Rio Grande do Norte	3568644 (1.69)	76.40	1.72
Sergipe	2331323 (1.11)	73.40	1.73
Nordeste	57883049 (27.48)	72.50	1.93
Brasil	210659013 (100%)	76.60	1.69
**Location**	IDH**	Unemployment rate**	Median per capita household income (R$)**
Alagoas	0.683	20.00	451.29
Bahia	0.714	21.30	484.90
Ceará	0.735	15.10	496.84
Maranhão	0.687	17.00	496.84
Paraíba	0.722	15.80	518.90
Pernambuco	0.727	21.30	515.01
Piauí	0.697	14.50	495.95
Rio Grande do Norte	0.731	15.50	573.43
Sergipe	0.702	20.90	485.88
Nordeste	0.71	18.60	483.93
Brasil	0.765	14.70	804.75

* The proportion of the population in relation to the Brazilian population. Source: Brazilian Institute of Geography and Statistics available at: https://sidra.ibge.gov.br/home/pms/brasil (2018 data).

** Source: Brazilian Institute of Geography and Statistics available at: https://sidra.ibge.gov.br/home/pms/brasil (2018 data).

Regarding health indicators, a higher proportion of the mammographies that were performed in the last two years, was observed among women aged 50 to 69 years, in the North-eastern states, that have better sociodemographic indicators. However, the rates observed in all North-eastern states were lower than 60% (observed proportion in Brazilian women) (Table 2). Conversely, there was a higher prevalence of women who had never had a mammogram, compared to what was observed in the Brazilian population (18.40%), ranging from 22.30% (Sergipe) to 41.90% (Maranhão) (Table 2).

**Table 2:** The health situation in the states of the Northeast region of Brazil.

**Location**	**Breast cancer incidence rate ^**1**^**	**% of women who have never had a mammogram ^**2**^**
Alagoas	37.04	24.10
Bahia	40.55	24.50
Ceará	50.54	38.60
Maranhão	27.18	41.90
Paraíba	46.17	31.40
Pernambuco	43.74	25.70
Piauí	35.01	36.10
Rio Grande do Norte	56.33	29.20
Sergipe	44.27	22.30
Nordeste	44.29	30.10
Brasil	61.61	18.40
**Location**	**% of mammography performed in the last 2 years** ^**2**^	**Number of oncology care services** ^**3**^
Alagoas	48.50	5
Bahia	57.90	14
Ceará	41.10	9
Maranhão	31.90	3
Paraíba	42.30	4
Pernambuco	51.00	10
Piauí	41.80	3
Rio Grande do Norte	47.60	7
Sergipe	52.10	2
Nordeste	47.90	57
Brasil	60.00	307
**Location**	**IFDM Health** ^**4**^	**Child mortality rate** ^**5**^
Alagoas	0.75	18.20
Bahia	0.59	20.50
Ceará	0.81	14.30
Maranhão	0.58	19.10
Paraíba	0.77	15.20
Pernambuco	0.82	15.70
Piauí	0.68	22.30
Rio Grande do Norte	0.79	15.20
Sergipe	0.76	19.90
Nordeste	0.64	16.40
Brasil	0.77	14.00

^1^Incidence rate per 100,000 women available in the 2020 Estimate Cancer Incidence in Brazil. Rio de Janeiro: INCA; 2020.

^2^Women aged 50 to 69 years—Source: IBGE National Health Survey 2013.

^3^Number of oncology care services registered with the SUS in 2018.

^4^Municipal Health Development Index (IFDM-Saúde/2016) state median available at https://www.firjan.com.br/ifdm/downloads/.

^5^Infant mortality rate per thousand live births in 2016 available at http://svs.aids.gov.br/dantps/centrais-de-conteudos/paineis-de-monitoramento/saude-brasil/mortalidade-na-infancia/.

The Brazilian Northeast Region is the second most populous geographic region in Brazil, however, it only has 18.56% of the oncology care network linked to the country’s SUS, which is concentrated in the Southeast (47.23%) and South (22.80%) regions (Table 2). The infant mortality rate represents an important indicator of socioeconomic conditions and access to health services. That said, there are high rates of this health indicator in the North-eastern states, being higher than the national coefficient, especially in the states of Piauí, Bahia, and Sergipe which presented the highest rates (Table 2).

The Municipal Development Index (IFDM), which assesses the health conditions of all municipalities in Brazilian states, is an indicator that groups the following items in its assessment: proportion of adequate prenatal care; proportion of registered deaths with ill-defined cause; infant deaths from preventable causes; and hospitalizations for causes sensitive to primary care, that is, complications of health problems that could be resolved in primary health care. An IFDM between 0.0 and 0.39 represents a low stage of socioeconomic and health development, an IFDM between 0.40 and 0.59 represents regular development, an IFDM between 0.60 and 0.79 moderate development, and an IFDM between 0.80 and 1.0 high stage of development. Seventy-eight percent of the states in the Northeast region showed moderate development, and only the states of Pernambuco and Ceará showed high socioeconomic and health development (Table 2).

### Study variables

The data used in this study were freely accessed from the Mortality Information System of the Informatics Department of the Unified Health System (*SIM/DATASUS*) on the website: http://www2.datasus.gov.br/DATASUS / [19]. There are no identified individuals in this system; therefore, this study was not submitted to a Research Ethics Committee. This is in accordance with national and international legislation that regulates research involving humans.

Population data for mortality rate estimates were also obtained from DATASUS (http://www2.datasus.gov.br/DATASUS/index.php?area=0206&id=6942) in the sociodemographic and economic data section, based on a demographic census from 1980, 1991, 2000 and 2010. The Brazilian Institute of Geography and Statistics estimated populations projections on July 10 of the intercensal years [20].

SIM/DATASUS is the information system of the Ministry of Health of Brazil, which provides death records for all Brazilian states and municipalities from 1979 to 2019. In the present study, the microdata of each state in the Northeast region for the years 1980 to 2019 was retrieved. The microdata is available in the dbc extension and was converted to the dbf extension through the Tabwin program version 4.15 for Windows provided by the Ministry of Health of Brazil. After converting the data to the dbf format, the death records of each year (1980 to 2019) were grouped for each of the states in the R software (version 4.1), extracting only female death records of females above the age of 20.

In the present study, we evaluated the following encodings as the underlying cause of death, the Ninth and Tenth International Classification of Diseases (ICD-9 and ICD-10) classifications: breast cancer (CC): 174 (ICD-9) and C50 (ICD-10); incomplete diagnosis of general cancer and incomplete (195, 196, 197, 198, 199, C76, C77, C78, C79, C79, C97, C76, C77, C80 and C97).

It is known that long-term disease breast cancer can trigger other diseases and health problems, which can be the basic cause of death, with breast cancer as an associated cause. However, Brazil and its regions present important problems in the quality and coverage of death records [21–23], with a high proportion of non-completion of information that is not mandatory (such as associated causes) in the death certificates [21–23], especially in locations with greater socioeconomic vulnerability. Thus, in this study, we chose to work with breast cancer as the underlying cause of death, because if it to include breast cancer as an associated cause, information bias could be introduced in the study, changing the temporal trend and temporal effects in breast cancer mortality.

There are significant disparities in quality of information and coverage of *SIM/DATASUS* death records between regions according to socioeconomic development. Between the 1990s and 2000s, there was a significant improvement in the information coverage and quality for all geographic regions of Brazil [21–23]. However, states in the northern and north-eastern regions with the lowest socioeconomic development still present significant problems in their Mortality Information Systems. Thus, in the present study, techniques were applied to correct these limitations [21–23].

The correction process was independently carried out by three authors, confirmed by a fourth author, and included the following steps: (i) proportional redistribution of 50% of deaths classified as ill-defined cause among defined natural causes [11] stratified by the north-eastern states; (ii) the proportional redistribution, according to age group and year, of deaths classified as incomplete diagnosis among all cancers; the proportional redistribution, by age group and year, stratified by north-eastern states; (iii) the sum of the values obtained in the previous steps was added to the breast cancer deaths registered in SIM/DATASUS; and (iv) finally, a correction in death coverage (underreporting), using the correction factors proposed by Queiroz et al. (2017) [21], for females according to the Brazilian states of the 1980s, 1990s, 2000s and 2010s. At this stage, the correction factors for each decade were multiplied by the number of deaths obtained in step iii. All the steps of the correction process were carried out in the R statistical program version 4.1.

When correcting the death records, we chose to work with age groups and periods grouped at five-year intervals. Age groups from 20–24 years to 80 years or older were evaluated due to excess zeros in smaller groups, resulting in *I* = 13 age groups, *J* = 8 time periods, and *K* = *I* + *J*– 1 = 20 birth cohorts, ranging from 1900 to 1994 [16–18]. Where i = 1, …, I; j = 1, … J; k = 1, …, K; and where K = I + J-1.

Breast cancer mortality rates, age group and geographic region per 100,000 women were calculated by 5-year age groups. Truncated rates for ages at open intervals (80 years and over) were calculated by year. After obtaining the rates by age groups and open ages intervals, the five-year periods were standardized by the direct method, using the standard population proposed by Segi (1966) and modified by Doll and Hill [24].

### Statistical analysis

The effects of Age, Period and Cohort (APC) on breast cancer mortality were estimated for each of the nine states in the Northeast region, considering the Poisson distribution of the number of deaths. The natural logarithm of the expected rate value is a linear function of age, period and cohort effects [16–18].

$$ln\left(E\left[r_{ij}\right]\right)=ln\left(\frac{\theta_{ij}}{N_{ij}}\right)=\mu +\alpha_{i}+\beta_{j}+\gamma_{k},$$

where E[r_ij_] represents the expected mortality rate at age *i* and period *j*; θ_ij_, number of deaths at age *i* and period *j*; N_ij_ denotes the population at risk of death at age *i* and period *j*; μ represents the average rate; α_1_ corresponds to the effect of age group *i*; β_j_, the effect of period *j*; and γ_k_, the effect of cohort *k*. The cases, yij, are specifed as the y-variate, Poisson errors with log link and ln(Nij) as an offset and then ot the factors age, period and cohort [17].

In the present study age, period and cohort (APC) effect parameters were estimated using the approach proposed by Holford, this method limits the effect analysis to its linear combinations and curvatures. The curvatures represent estimable functions of the parameters and make them constant, despite the parameterization used [16–18]. In addition, the linear trend of effects is divided into two components: the first is the linear effect of age and the other is called drift, the linear effect of period and cohort. The sum of the age and period slopes (α_L_+β_L_) constitutes the longitudinal trend of age, where α_L_ and *β*_*L*_ are linear trend of age and period respectively, whereas the linear trend of the age-specific rates logarithm represents that the drift term is equal to the sum of the period and cohort slopes (β_L_ + γ_L_), where β_L_ and *γ*_L_ are the linear trend of period and cohort, respectively [16, 17].

In the present study, the period from 1995 to 1999 was the reference period, the reference cohort was that of 1945–1949, because the central cohorts tend to be more stable and complete than the first and last cohorts [16, 17].

The quality of fit was assessed using deviance statistics, defined as two times the logarithm of the estimated likelihood function of the model. The contribution of the effects was assessed by comparing the deviance of the model with the specifc effect compared to the complete model (age-period-cohort). Results with p ≤ 0.05 were considered significant. Formal testing of the effects done through a sequence of relevant sub-models conveniently arranged, allowing to compare models between adjacent lines (Table 3).

**Table 3:** Sequential structure of model comparison.

Model	Log[λ(a,p)]
Age	*f*(*a*)
Age-*drift*	*f*(*a*)+*δ*c *
Age-Cohort	*f*(*a*)+*h*(*c*)
Age-Period-Cohort	*f*(*a*)+*g*(*p*)+*h*(*c*)
Age-Period	*f*(*a*)+*g*(*p*)
Age-drift	*f*(*a*)+*δ*p *

^*^linear trend of the logarithm of age-specific rates, which is equal to the sum of the of period and cohort slopes (βL + γL), where βL and γL are the linear trends for the period and cohort, respectively.

^**^longitudinal trend of age is the sum of age and period slopes (αL + βL), where αL and βL are the linear trends of age and period, respectively.

The risk of death was estimated by relative risk (RR) estimates and 95% confidence intervals according to period and cohort effects. Estimates for the Age Period and Cohort models were made using the Epi library 1.1.18 (R Foundation of Computational Statistics, Vienna, Austria http://www.r-project.org) of the R program (version 4.1) [25].

## Results

In the period of 1980 to 2019 in the Northeast of Brazil, 65,531 deaths due to breast cancer were recorded in women aged 20 years and over (13.28 deaths per 100,000 women). After the correction process, there was an increase of 54.15% in the death records, representing a mortality rate of 20.45 deaths per 100,000 women. After this correction of the death records, there was an increase of about 22.0% in mortality rates in all Northeast states, varying from 22.3% in Pernambuco to 87.18% in Maranhão. The states that showed the greatest increases in the average mortality rates after the correction process of the death records were Maranhão (87.18%), Paraíba (41.64%), and Piauí (39.97%). In the other locations, the percentage increase was around 36%, with the exception of Sergipe (28.19%).

In all of the locations under study, a significant percentage increase in breast cancer mortality rates was observed when comparing the first five-year period (1980–1984) to the last five-year period (2015–2019). This increase was over 60%, varying from 62.0% in Alagoas to 222.00% in Maranhão (Table 4).

**Table 4:** Breast cancer mortality standardized rates (per 100,000 women) from 1980 to 2019 in the states of north-eastern Brazil.

Locality	Mortality rates	Period							
Northeast		1980–1984	1985–1989	1990–1994	1995–1999	2000–2004	2005–2009	2010–2014	2015–2019
	UMR^a^	7.16	7.86	8.69	9.68	11.33	14.95	16.87	18.76
	CMR^b^	10.58	11.47	12.25	13.05	14.55	17.16	18.91	19.70
	CMRQIUD^c^	14.70	15.95	15.92	16.97	18.63	21.96	24.20	25.22
Alagoas	UMR	7.95	7.23	7.71	7.15	8.99	13.71	15.88	16.10
	CMR	12.07	10.79	11.15	10.28	11.98	15.96	18.00	17.50
	CMRQIUD	12.31	11.03	11.56	10.67	13.70	18.20	20.53	19.95
Bahia	UMR	7.77	8.76	8.97	9.41	10.50	13.12	16.08	17.60
	CMR	10.76	11.70	11.75	12.23	13.36	15.57	18.63	20.16
	CMRQIUD	11.85	12.87	13.52	14.05	15.50	18.06	21.62	23.39
Ceará	UMR	7.41	7.92	8.55	11.51	13.99	17.05	18.30	21.82
	CMR	10.45	11.58	12.50	15.11	18.14	19.62	20.33	23.61
	CMRQIUD	13.60	15.06	14.86	17.98	20.68	22.38	23.17	26.92
Maranhão	UMR	2.34	3.14	3.53	3.91	4.33	8.38	10.89	12.11
	CMR	3.39	4.91	5.51	5.66	6.09	9.35	11.89	13.20
	CMRQIUD	6.39	9.28	8.39	8.60	9.50	14.59	18.55	20.59
Paraíba	UMR	5.40	5.25	6.59	6.07	8.51	15.16	15.66	16.84
	CMR	10.79	9.69	11.26	10.04	12.25	17.58	17.65	18.25
	CMRQIUD	11.22	10.08	11.98	10.69	13.70	19.70	19.74	20.92
Pernambuco	UMR	10.01	10.94	12.77	15.16	16.40	19.68	20.19	22.19
	CMR	14.73	15.87	17.54	18.75	19.72	22.01	22.24	23.96
	CMRQIUD	14.76	15.88	17.90	19.13	20.70	23.09	23.36	25.16
Piauí	UMR	5.42	3.78	5.09	4.84	8.62	13.32	15.93	17.80
	CMR	7.75	5.86	7.18	6.34	10.94	14.73	17.25	18.75
	CMRQIUD	13.84	10.38	8.53	7.51	12.71	17.09	19.99	22.15
Rio Grande do Norte	UMR	5.74	9.52	10.63	10.97	11.51	15.05	17.53	20.07
	CMR	8.03	13.18	14.35	14.52	14.57	17.24	19.75	22.09
	CMRQIUD	8.68	14.41	16.11	16.23	16.89	20.00	22.88	25.63
Sergipe	UMR	7.80	8.55	9.48	9.64	13.49	17.44	20.94	22.25
	CMR	12.24	13.61	14.16	13.63	16.62	19.17	22.58	23.99
	CMRQIUD	12.58	13.84	14.94	14.50	18.25	21.08	24.87	26.38

^**a**^Uncorrected mortality rates (UMR)^;^

^b^CMR Death correction: ill-defined cause among defined natural causes and deaths classified as incomplete diagnosis among all cancers;

^c^CMRQIUD- All steps of correction of mortality rates were corrected for ill-defined causes, unspecified uterine cancer, incomplete cancer and underreporting of death.

**Fig 1:**
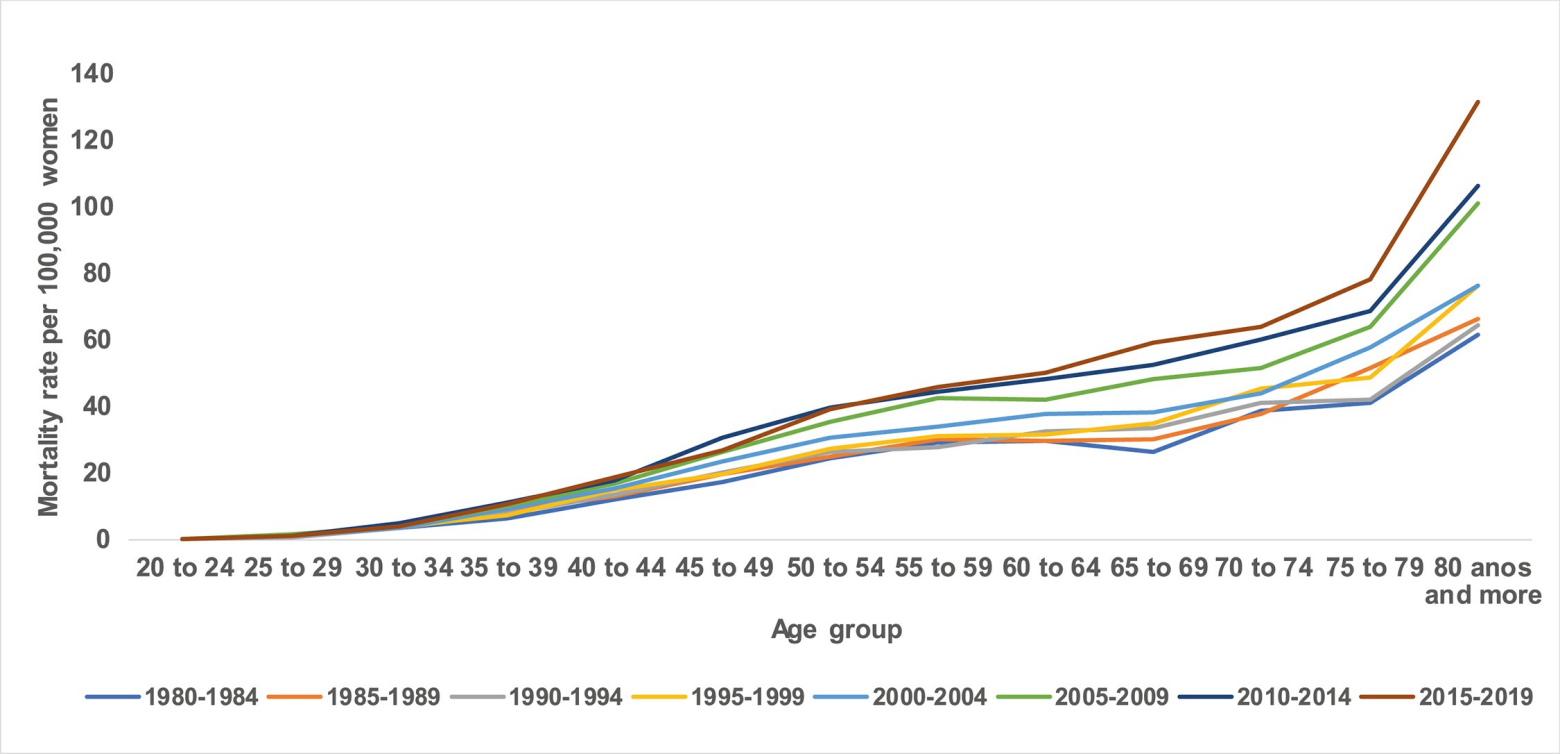
Breast cancer mortality rates by age group and death period in Northeast Brazil, 1980–2019.

**Fig 2:**
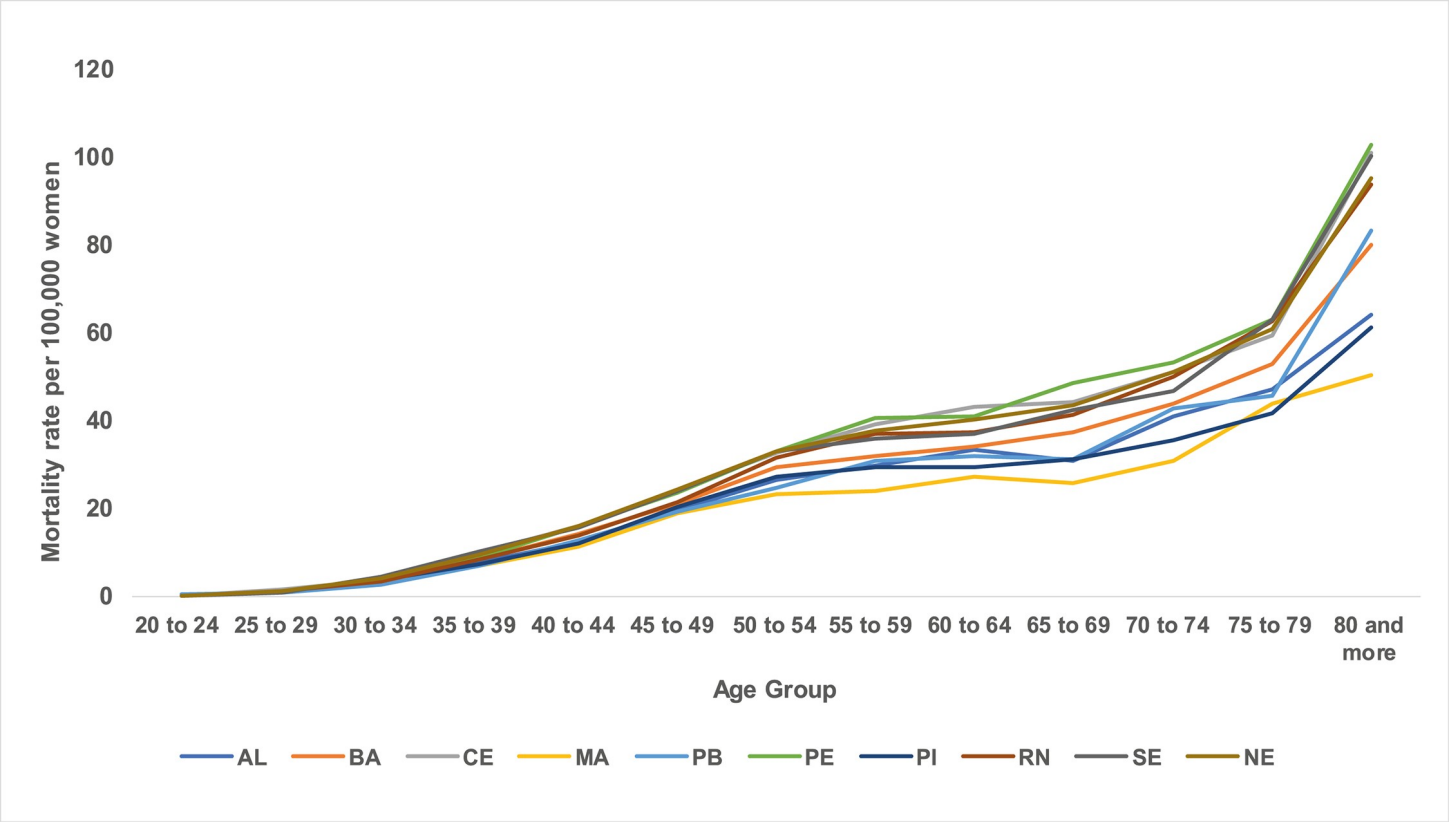
Distribution of mean mortality rates observed for breast cancer according to age groups in Northeast states, Brazil, 1980–2019.

The states with the highest mortality rates were Pernambuco (21.09 deaths) and Ceará (20.85 deaths), and the lowest mortality rates were Maranhão (13.58 deaths) and Piauí (15.43 deaths) within the stipulated periods, with the highest rates being presented in the five-year period of the 2000s (Fig 1). In all states in the Northeast region, there was a progressive increase in mortality rates with advancing age, with the lowest coefficients observed in the 20–24 age group and a progressive increase with advancing age, with the highest rates being presented in the age group of 80 years and over. Disparities are observed between the magnitude of mortality rates in the Northeast states. The lowest coefficients were found in the states of Piauí and Alagoas, and the highest in the states of Pernambuco and Ceará, especially from the age of 50 years onwards (Fig 2).

**Fig 3:**
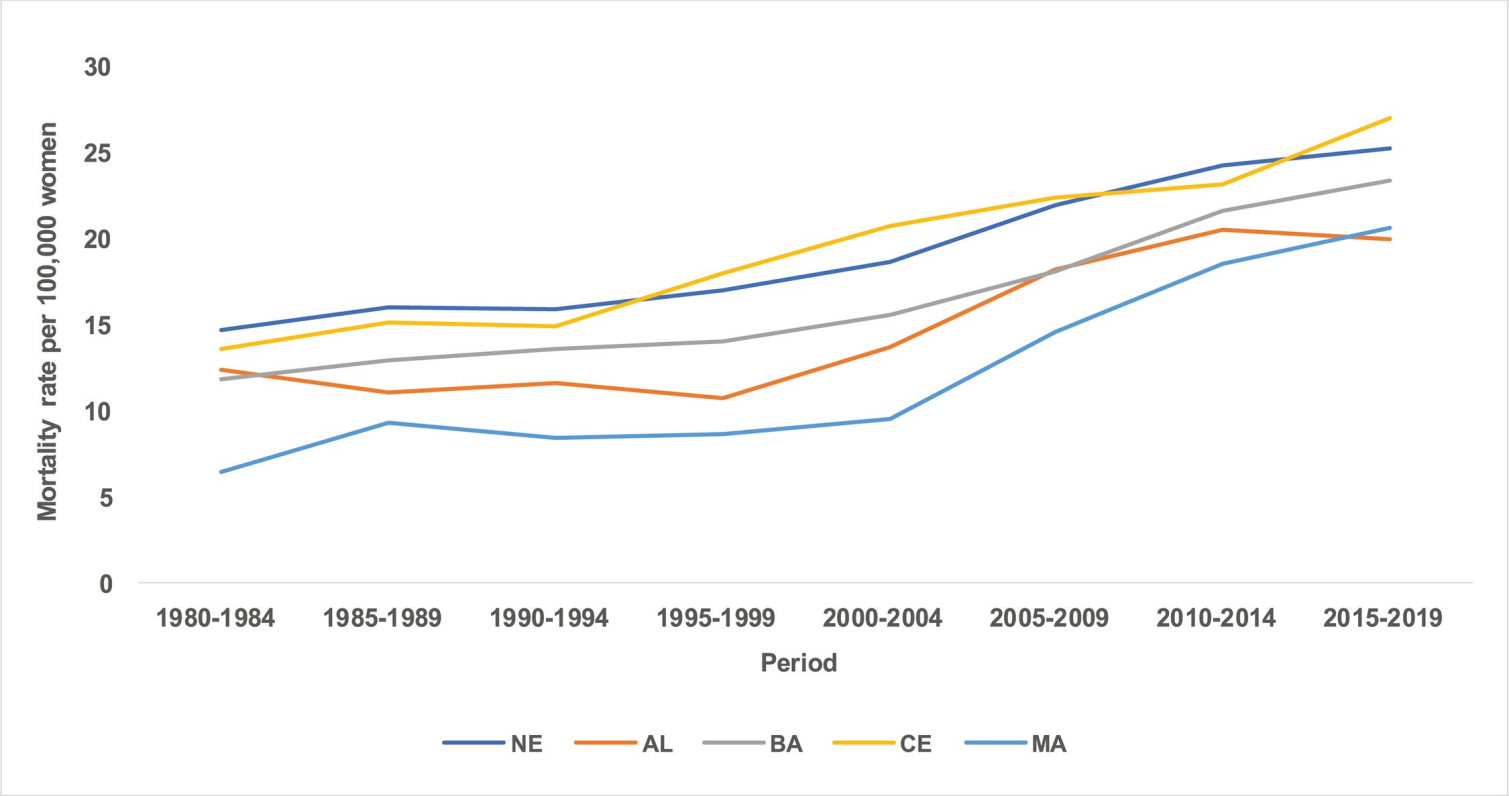
Mortality rates for breast cancer in the Northeast states of Brazil, by period 1980 to 2019.

**Fig 4:**
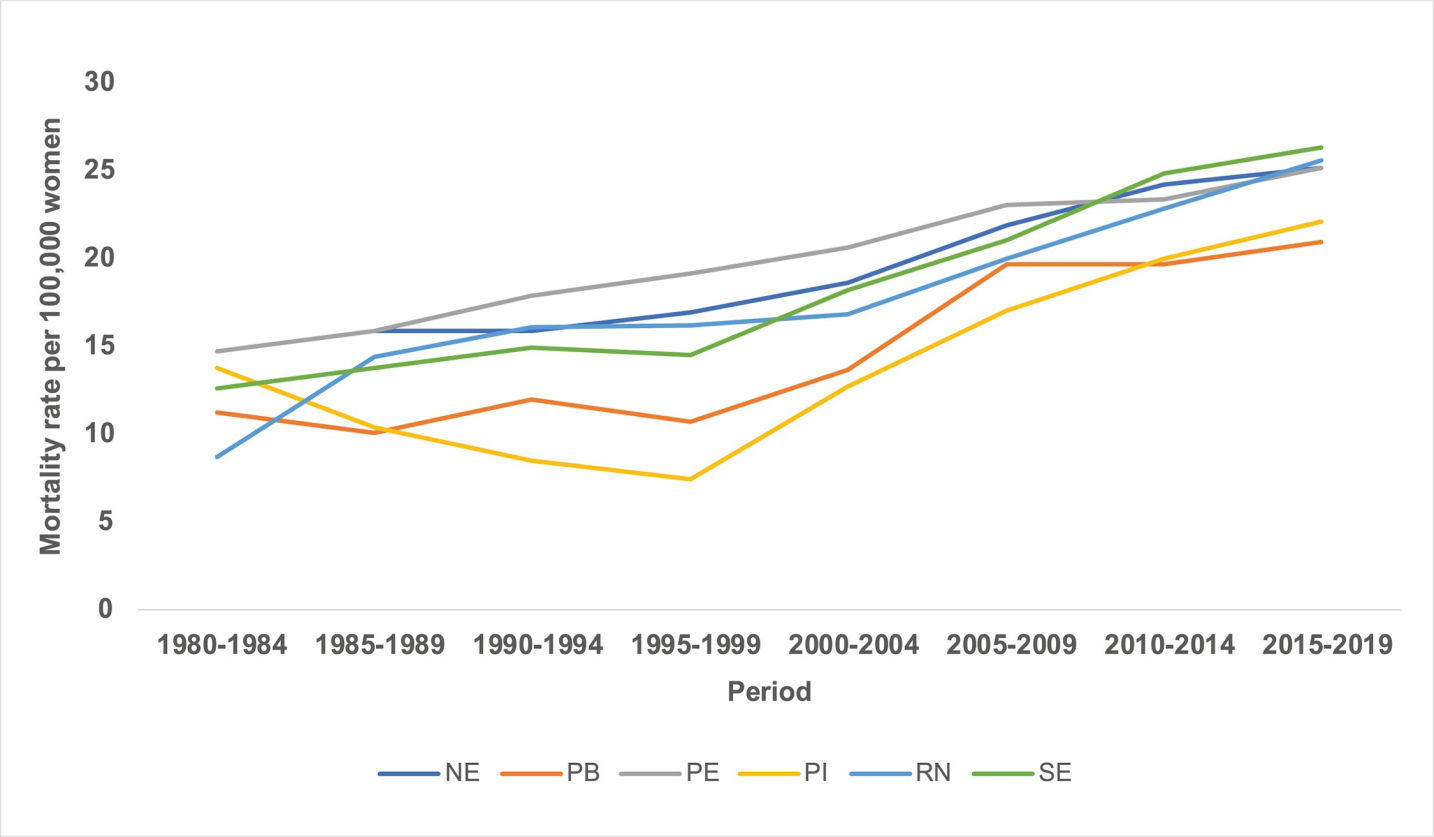
Mortality rates for breast cancer in the Northeast states of Brazil, by period 1980 to 2019.

There was an increase in breast cancer mortality rates between the five-year periods from 1980 to 2019, with higher rates presented in the five-year period of the 2000s (Figs 3 and 4).

**Fig 5:**
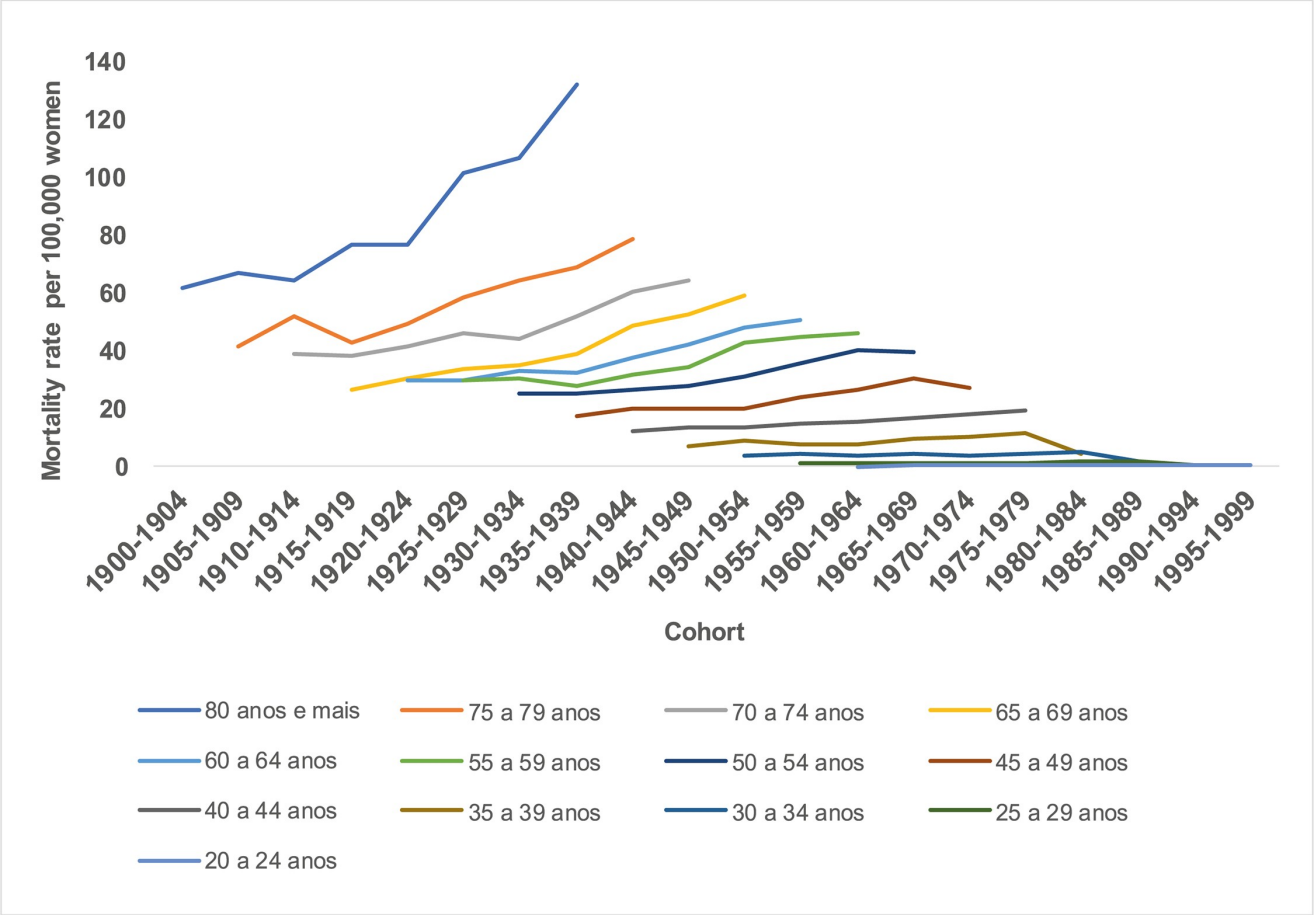
Breast cancer mortality rates by age group and death cohort in Northeast Brazil, 1980–2019.

**Fig 6:**
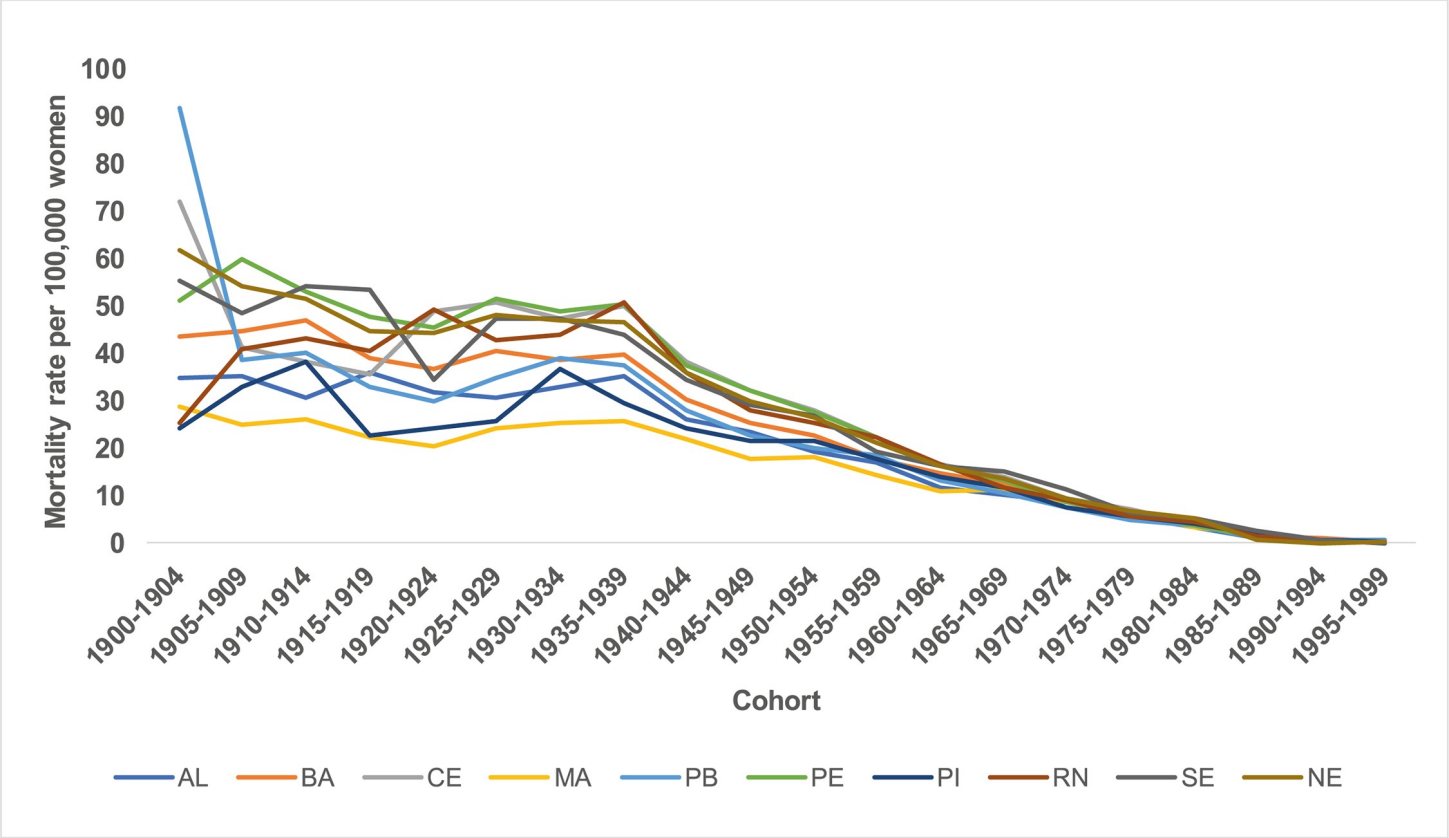
Distribution of mean mortality rates observed for breast cancer according to cohort groups in Northeast States, Brazil, 1980–2019.

An upward temporal trend was observed in the cohorts of 1900 to 1904 (80 years and older) to 1965 to 1969 (45 to 49 years), with a slight reduction from the 1970 cohort (35 to 44 years). Thus, women residing in the Northeast region, as well as in each of its states, presented higher mortality rates among women from older generations, (Figs 5 and 6).

After estimating the Age, Period and Cohort models, it was found that the Northeast region and all its states presented the complete model (APC) as the best fit model for the data (assessed using deviance statistics and p-value), with the exception of Pernambuco, Rio Grande do Norte, and Sergipe, in which the models of best fit were age-cohort and age-drift (sum of the period and cohort slopes (βL + γL), respectively, where βL and γL represented the linear trends for the period and cohort) (Table 5).

**Table 5:** Deviance an p-value analysis in sequential construction of age, period and cohort models.

Locality			
	Northeast		
Models	Df^a^	Residual Deviance	p (>|Chi|)
Age	96	6100.4	
Age-drif^t^^b^	95	2580.9	<0.0001
Age-Cohort	89	3519.4	<0.0001
Age-Period- Cohort	83	2396.6	<0.0001
Age-Period	89	2221.8	0.004
Age-drift^c^	95	2580.9	<0.0001
	Alagoas		
Models	Df^a^	Residual Deviance	p (>|Chi|)
Age	96	369.49	
Age-drif^t^^b^	95	153.65	<0.0001
Age-Cohort	89	139.38	<0.0001
Age-Period- Cohort	83	85.22	<0.0001
Age-Period	89	104.21	0.004
Age-drift^c^	95	153.65	<0.0001
	Bahia		
Models	Df^a^	Residual Deviance	p (>|Chi|)
Age	96	1973.80	
Age-drif^t^^b^	95	729.74	<0.0001
Age-Cohort	89	711.43	0.00038
Age-Period- Cohort	83	654.38	<0.0001
Age-Period	89	681.54	<0.0001
Age-drift^c^	95	729.74	<0.0001
	Ceará		
Models	Df^a^	Residual Deviance	p (>|Chi|)
Age	96	1311.18	
Age-drif^t^^b^	95	576.47	<0.0001
Age-Cohort	89	494.05	<0.0001
Age-Period- Cohort	83	475.56	0.000348
Age-Period	89	564.21	<0.0001
Age-drift^c^	95	576.47	<0.0001
	Maranhão		
Models	Df^a^	Residual Deviance	p (>|Chi|)
Age	96	1228.59	
Age-drif^t^^b^	95	333.77	<0.0001
Age-Cohort	89	327.50	0.09943
Age-Period- Cohort	83	265.97	<0.0001
Age-Period	89	274.20	0.004
Age-drift^c^	95	333.77	<0.0001
	Paraíba		
Models	Df^a^	Residual Deviance	p (>|Chi|)
Age	96	2430.8	
Age-drif^t^^b^	95	1775.5	<0.0001
Age-Cohort	89	1593.9	0.004
Age-Period- Cohort	83	1348.5	<0.0001
Age-Period	89	1578.7	<0.0001
Age-drift^c^	95	1775.5	<0.0001
	Pernambuco		
Models	Df^a^	Residual Deviance	p (>|Chi|)
Age	96	1059.90	
Age-drif^t^^b^	95	576.21	<0.0001
Age-Cohort	89	482.23	<0.0001
Age-Period- Cohort	83	478.16	0.25452
Age-Period	89	567.63	<0.0001
Age-drift^c^	95	576.21	0.03548
	Piauí		
Models	Df^a^	Residual Deviance	p (>|Chi|)
Age	96	1059.90	
Age-drif^t^^b^	95	576.21	<0.0001
Age-Cohort	89	482.23	<0.0001
Age-Period- Cohort	83	478.16	0.25452
Age-Period	89	567.63	<0.0001
Age-drift^c^	95	576.21	0.03548
	Rio Grande do Norte		
Models	Df^a^	Residual Deviance	p (>|Chi|)
Age	96	655.41	
Age-drif^t^^b^	95	297.81	<0.0001
Age-Cohort	89	268.71	0.001
Age-Period- Cohort	83	294.89	0.1276
Age-Period	89	294.89	<0.0001
Age-drift^c^	95	297.81	0.4038
	Sergipe		
Models	Df^a^	Residual Deviance	p (>|Chi|)
Age	96	417.85	
Age-drif^t^^b^	95	195.60	<0.0001
Age-Cohort	89	194.91	0.8767
Age-Period- Cohort	83	189.13	0.1230
Age-Period	89	189.95	0.8447
Age-drift^c^	95	195.60	0.1304

^a^Degrees of freedom.

^b^linear trend of the logarithm of age-specific rates, which is equal to the sum of the of period and cohort slopes (βL + γL), where βL and γL are the linear trends for the period and cohort, respectively.

^c^longitudinal trend of age is the sum of age and period slopes (αL + βL), where αL and βL are the linear trends of age and period, respectively.

**Fig 7:**
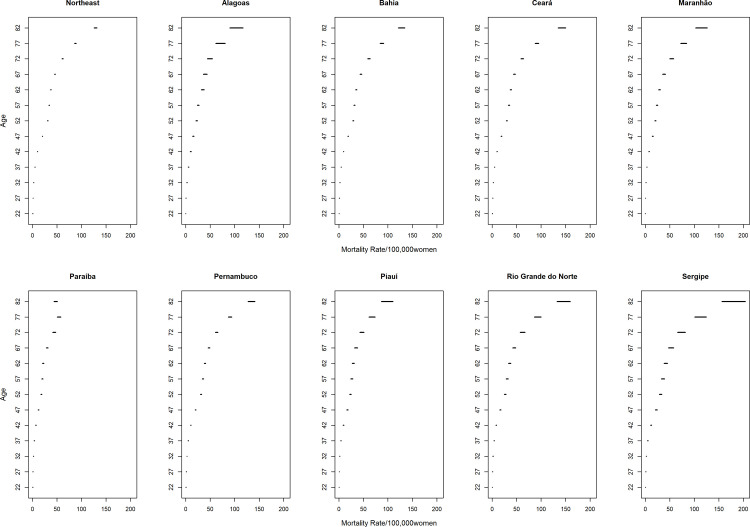
Results of the age-period-cohort model adjusted for breast cancer mortality according to the age effect and states of the Northeast, Brazil, 1980–2019.

With regard to the temporal effect of age after adjusting the APC models, there was a progressive increase in average mortality rates with increasing age, confirming the findings of the exploratory analysis. However, the highest percentage increases between age groups were seen in ages up to 44 years, being over 100%, and the most advanced age groups presented the lowest percentages. The age groups between 55–59 years and 60–64 years stood out, presenting values equal to 8% and 10%, respectively, in relation to their immediate previous ranges (Fig 7).

**Fig 8:**
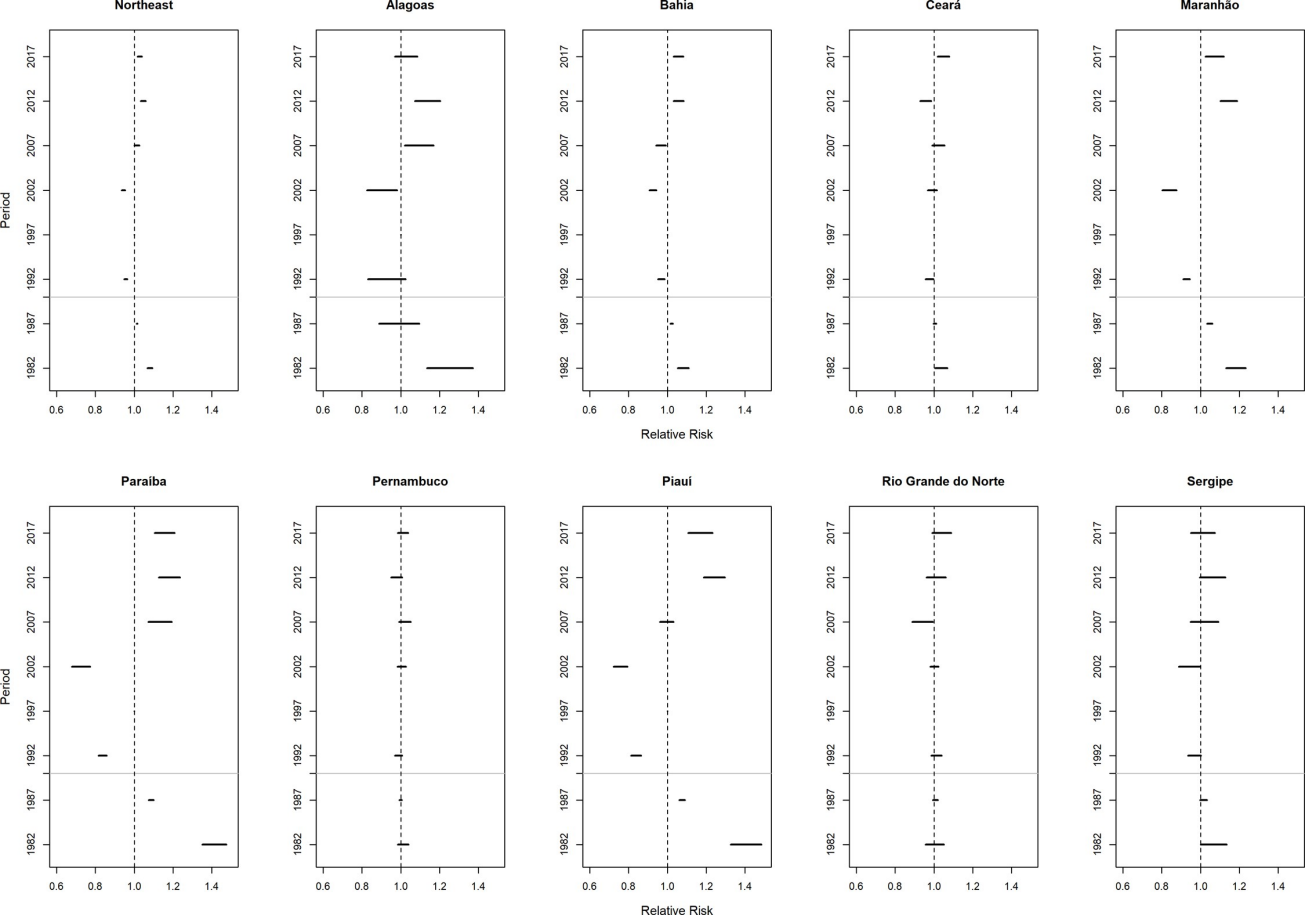
Results of the age-period-cohort model adjusted for breast cancer mortality according to the period effect and states of the Northeast, Brazil, 1980–2019.

Regarding the effect of the period, there was heterogeneity between the locations under study. In the Northeast region, there was an increased risk of death (RR> 1) from breast cancer in relation to the reference period (1995–1999) in the 1980s (1980–1984 to 1985–1989) and in the five-year periods of 2005–2009, 2010–2014 and 2015–2019. There was a reduction of the risk of death in the following five-year periods: 1990–1994 and 2000–2004. Similar results were observed in the states of Alagoas, Bahia, Maranhão, Paraíba, and Piauí (Fig 8). In Ceará, there was a reduction in the risk of death in the period of 1990 to 2014, with an increase in the last five-year period (2015–2019). In Pernambuco, there was no significant period effect, and in the states of Rio Grande do Norte (2005–2009) and Sergipe (2000–2004) there was a statistically significant reduction in only one period (Fig 8).

**Fig 9:**
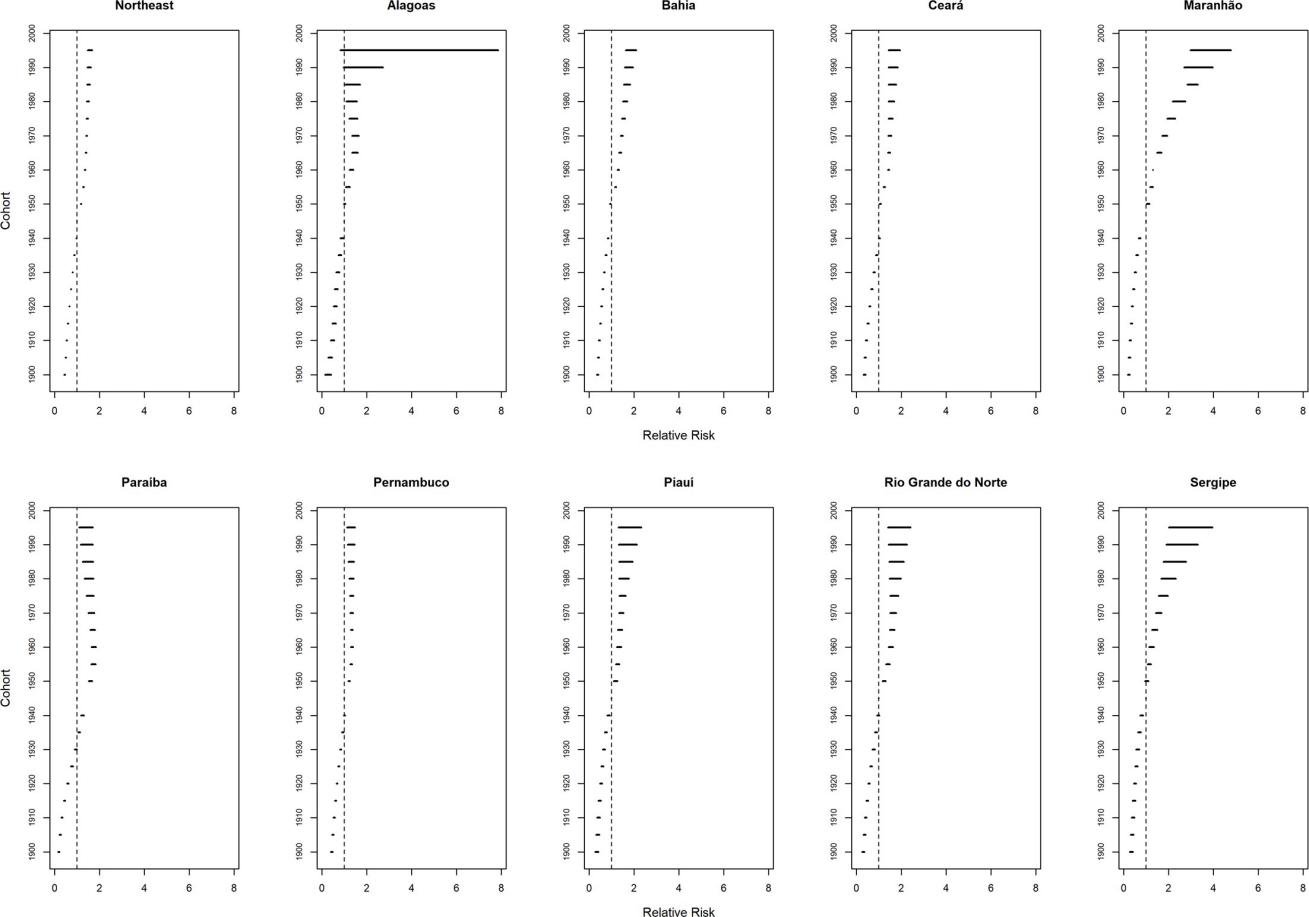
Results of the age-period-cohort model adjusted for breast cancer mortality according to the cohort effect in north-eastern states, Brazil, 1980–2019.

Women living in the Northeast region who were born until the cohort from 1940 to 1944 had a lower risk of death from breast cancer (RR <1) when compared to the cohort from 1945 to 1949. Conversely, women born in the 1950s presented a progressive increase in the risk of death from this neoplasm (RR> 1) (Fig 9).

## Discussion

The temporal evolution of mortality from diseases and health problems is influenced by access to health services, diagnostic innovations, and improvements in the quality and coverage of death records. In Brazil, after the implementation of the Unified Health System (SUS), there was an increase in access to health services, especially in the 2000s, contributing to the improvement in the quality and coverage of death records [16–18].

The improvement in the quality and coverage of death records did not occur uniformly in all geographic regions. The states of the Northeast region still have a high proportion of records classified as ill-defined cause, incomplete cancer diagnosis, and low death coverage [26]. After the correction of the death records in four stages in the present study, there was an increase of more than 50% in breast cancer mortality rates, with higher percentages in the states with the worst conditions regarding access to health services (Maranhão and Piauí) in the 1980s and 1990s. This confirmed the need to apply indirect techniques to correct deaths in research in which SIM records are used, comparing different locations over a long period of time, especially in Brazilian locations with greater socioeconomic vulnerability [21–23].

The states of the Northeast region with the best socioeconomic and health indicators (Bahia, Ceará, and Pernambuco) showed higher breast cancer mortality rates as compared to the states with the greatest socioeconomic vulnerability (Maranhão and Piauí), the opposite was verified in the coefficients of mortality from cervical cancer [26]. A similar situation was observed in the incidence estimates for the 2020/2021 biennium [27], and when comparing the incidence and mortality rates for these cancers, and when comparing developed and developing countries [1].

The breast cancer mortality rate in the Northeast region in the 2000s is similar to the average mortality rates observed in Brazil in the period of 1980 to 2009 (22.30 deaths per 100,000 women), however, it was lower than the rate presented in the South region with the highest HDI in Brazil (30.0 deaths per 100,000 women) [12, 28]. In addition, the behavior of temporal trends diverged by region, with the pattern increasing in the North, Northeast, and Midwest and decreasing in the South and Southeast. It is noteworthy that the opposite was observed when analyzing mortality from cervical cancer in the states of the Northeast region [26] confirming the findings of Guimarães et al. (2016), in which higher breast cancer mortality rates were noted in the municipalities with the highest HDI and the highest coefficients for cervical cancer in the municipalities with the lowest HDI [29].

The reality that can correlate with the transition from cancer is that there is a relationship between the human development index (HDI) and cancer. Locations with a higher HDI have higher rates of cancer incidence associated with the westernization of habits and lifestyles, while locations with a lower HDI have higher coefficients of neoplasms associated with infection [1]. Another factor correlated with the differences in the profile of cancer incidence and mortality between these regions is the heterogeneity in the demographic transition. In Brazil, the demographic transition presented an accelerated pace when compared to developed countries, but with different paces between the different locations. In the states of the Northeast region, the transition was less accelerated, contributing to its less aged age structure in the country. It is highlighted that the states of Maranhão, Piauí, and Alagoas have a younger age structure and a high fertility rate, factors that may contribute to the lower burden of diseases associated with population aging in their epidemiological profile [30].

In this sense, the present study showed a progressive increase in the estimated mortality rates with advancing age in all states, with greater magnitude from 65 years of age onwards. Similar results were observed in Mexico, Spain, Taiwan, Germany, and Brazil [12, 31–34]. An increase in the risk of death from breast cancer is expected with advancing age since it is a chronic non-communicable disease associated with exposure to risk factors throughout life, with an increase in incidence from the fourth decade of life onwards, after menopause, with a peak incidence from the sixth decade of life [9, 12, 35].

However, it is essential to highlight that, from the youngest ages up to 44 years, the percentage increases in mortality rates were higher than 100%, while in the more advanced age groups, the magnitude of this growth was less intense, highlighting the age groups between 55–59 and 60–64 years in which reduced percentages and less than 20% were observed. This phenomenon has been verified in other studies and is known as the “Clemmensen hook”, both in incidence and in mortality rates [31, 36–38]. Some authors state that it is correlated with the overlapping of the temporal evolution of breast cancers in pre-menopausal and post-menopausal women [31, 36–38]. However, others believe that this phenomenon occurs due to changes in habits and lifestyles among the younger and older cohorts, and that it will possibly disappear in the coming decades [39, 40].

The temporal evolution of breast cancer mortality rates in the Northeast region in all its states showed a significant increase, especially in the five-year period of the 2000s. The increase in breast cancer mortality rates over the last 40 years (1980–2019) in the Northeast states is similar to that observed in developed countries in the period of 1990 to 2013, in which a higher proportional increase was observed in developing countries (46%) in relation to developed countries (8%), although mortality rates were higher in the latter [41]. Similarly, there has been an increase in the cumulative risk of mortality which has increased in Central American countries, in some of East Asia, North Africa, the Middle East, Eastern Europe, and sub-Saharan Africa. However, in some developing countries, such as India and China, this risk has decreased during the 1980–2010 period [42]. They evaluated the motivating factors for these trends and suggested that the reduction may have occurred due to early detection by mammography and improvements in treatment. The authors showed that divergent behavior was observed in countries like Brazil, Colombia, Ecuador, Egypt, Guatemala, Japan, Kuwait, Mauritius, Mexico, and Moldova [41, 43].

Regarding the period effect in the Northeast of Brazil, there was an increased risk of death from breast cancer in the states of Alagoas, Bahia, Maranhão, Paraíba, and Piauí in the quinquennia of the 2000s (2000 to 2019), and in Ceará (2015–2019) in relation to the reference period (1995–1999).

It is known that there are differences in the factors correlated to the period effect on the temporal evolution of breast cancer incidence and mortality. Changes in reproductive behavior (reduction in fertility rates, increased prevalence of pregnancy after the age of thirty, low prevalence of breastfeeding, among other factors), the westernization of habits and lifestyles, and access to mammographic examination have been related to the increased incidence of breast cancer in developed and developing countries [8–10, 31, 44, 45]. On the other hand, it is argued that access to health services for early detection (screening by means of mammography), timely treatment of the disease, and therapeutic innovations (hormone therapy, immunotherapy, and monoclonal antibody), which have been amplified since the 1990s, relate to the period effect of breast cancer mortality [9, 12, 31, 45–55]. These factors are mainly responsible for the reduction in the risk of death observed in developed countries, despite higher incidence rates in relation to developing countries [9, 10, 44, 45].

The increased risk of death in the most recent periods observed in most states in the Northeast is similar to those observed in Mexico, Russia, Ukraine, and Brazil and Brazilian´s geographic regions [12, 31, 48]. However, it differs from those presented by Japan, Singapore, South Korea, Spain, and Sweden, where the increased risk of incidence in more recent periods is accompanied by a reduction in the risk of death from breast cancer [8, 9, 31, 44, 45].

It is expected that, at the beginning of the screening programs, there will be an increase in mortality, since many women who were not previously exposed to secondary prevention (mammography) will be diagnosed in advanced stages of the disease, which reduces their survival and increases the quality of death records. With the consolidation of the screening program, a reduction in mortality is observed due to the increase in the proportion of women diagnosed in the early stages of the disease and the reduction in the proportion of diagnoses in advanced stages [56, 57].

In countries where there has been a reduction in the risk of death from breast cancer since the 1990s, population-based screening programs with coverage above 70% are observed, associated with timely treatment and access to therapeutic innovations in diagnosis, drug treatment (such as new chemotherapy protocols, hormone therapy, monoclonal antibody) and radiotherapy [8, 9, 31, 44, 45].

The globalization process has generated social, cultural, and economic impacts that have promoted health risks at different intensities between regions of the world [58, 59]. Low- and middle-income countries have experienced intense changes in their habits and lifestyle (Westernization of habits and lifestyles), increasing the prevalence of risk factors for chronic diseases in their population, which are responsible for the increase in incidence and mortality, especially due to cardiovascular disease and cancer. The inequalities observed in the trend of breast cancer mortality between developed and developing countries are also verified within the countries themselves, as access to early detection and timely treatment is highly correlated with access to health services and therapies [58–61].

Regions with greater socioeconomic vulnerability are unable to provide their population with the available therapeutic resources, contributing to the maintenance of an epidemiological profile in which neglected tropical diseases are associated with an increased prevalence of non-communicable chronic diseases. Disparities are observed between countries in the world and within each country, especially in locations that are excluded from the central circuits of the global economy [58–61]. A reality that points to the need to implement global health in which countries make efforts so that no individual or community is excluded from access to health care, nor to new diagnostic and therapeutic technologies [60, 61]. However, in recent years there has been an increase in socioeconomic disparities and in the implementation of fiscal adjustment in several countries, with a reduction in financing for social security, with a greater impact on the health of the low-income population [62], and with this, it is estimated that disparities in breast cancer mortality between regions of high and low socioeconomic vulnerability remain.

In Brazil, since the beginning of the 2000s, the Ministry of Health’s prevention and control program has recommended an annual clinical breast examination for women over 40 years old and biannual screening by means of mammography for women aged 50 to 69 years. These guidelines were revised in 2015 [63], thus maintaining these recommendations. The National Oncology Care Policy also came into force in 2005 and aims at expanding access to cancer treatment on a national level, encompassing actions ranging from primary prevention, through early detection, timely treatment, and palliative care [64]. Despite the advances achieved in the prevention and control of cancer in the last fifteen years, important regional inequities still persist in the Brazilian cancer care network, with a large concentration of mammographs, the Oncological Prevention Center, and radiotherapy devices in the South and Southeast regions of Brazil [13, 63].

It is noteworthy that the Northeast region presented a relationship between mammography devices and women well below that recommended by the WHO (50 mammograms for every 100,000 women) [15]. Thus, women living in the Northeast region are less likely to be screened for breast cancer, increasing the risk of diagnosis in advanced stages. This is also related to the smaller network of cancer care facilities present in these states, contributing to the increase in mortality observed in these states, especially in the quinquennia of the 2000s [12, 15, 64, 65].

Access to screening and timely treatment with therapeutic innovations can influence the temporal evolution of breast cancer mortality in successive cohorts. In developed countries, an increase in incidence was observed, especially in younger cohorts, due to changes in the reproductive behavior of women, with a high proportion of pregnancies after the age of 30, the option of not being pregnant, a high prevalence of the use of oral contraceptives, and hormonal replacement therapies [63–68]. These changes were accompanied by the progressive prevalence of fat consumption, sedentary lifestyles, and increased alcohol intake in the younger cohorts [6, 67–72]. However, the increased incidence in younger cohorts and in more recent periods was not accompanied by an increased risk of death in those same generations and periods, correlating with greater access to screening and therapeutic innovations mentioned earlier in this discussion, and thus contributes to early diagnosis and timely treatment, consequently increasing survival [9, 31, 44–46].

In Brazil, with the exception of the Southeast region and the city of São Paulo, an increased risk of death was observed for the younger cohorts [12, 73], corresponding to what was observed in the present study. Women living in the Northeast states who were born after the 1950 cohort exhibited greater risks of death from breast cancer when compared to those of the 1945–1949 generation, with an increasing trend in all states. Since the cohort effect was not statistically significant for inclusion in the statistical model for the results from Sergipe, the cohort effect was estimated by the age model with the linear combination of age and period. This showed an increased risk for the younger cohorts.

The results obtained for the cohorts are corroborating other studies that showed an increased risk of death in younger cohorts in developing countries [31, 74, 75]. These results may be correlated with the increased incidence in women of younger generations, as they are more exposed to the main risk factors for breast cancer, as previously mentioned [8, 9, 31, 44–46]. The increased risk of incidence in the younger cohorts in the Northeast region of Brazil was not accompanied by an expansion of the coverage of mammography and of the network of sufficient cancer care to promote a reduction in the diagnosis of the disease in advanced stages and, thus, increase survival.

In the United Kingdom, the reduction in fertility rates was correlated with the increased risk of cancers related to reproductive factors (breast, ovary, and endometrium) [3]. The increased risk of breast cancer mortality was related to reduced fertility levels in US cohorts [76]. In Korea, 16.7% of breast cancer cases were attributed to reproductive factors such as late maternal age, non-breastfeeding, the use of oral contraceptives, and the use of hormone replacement therapies [6]. In Brazil, these fractions were 2.1% and 1.4% for the use of oral contraceptives and non-breastfeeding, respectively [5].

The total fertility rate in Brazil ranged from 7.1 children per woman in the first decade of the 20th century to 1.9 children in 2010 [73]. Nulliparity has also registered an increase in the country and, among the highlighted micro-regions, these cases were located in Pernambuco [77]. Late maternal age has also been evidenced as a change in the reproductive behavior of Brazilian women in recent decades [78, 79].

A study on the temporal evolution of total fertility rates estimated for 17 cohorts of Brazilian women born between 1890 and 1975 showed a reduction of 6.2 children among women born in 1890–1895 to 2.5 children in the 1970–1975 cohort. Fertility showed a generalized tendency of reduction in all regions, mainly for women born between 1940–1945, but with a different rhythm between geographic regions. In the Northeast, this trend was more accelerated from the cohort born between 1950–1955 [80], and the risk of death from breast cancer showed similar behavior in the present study.

In the Northeast region, the process of declining the total fertility rate started in the early 1970s, dropping from 7.53 children per woman to 3.12 children in 1996 [81]. Between 1970 and 2010, the most significant declines were seen in the states of Pernambuco and Rio Grande do Norte and the least accentuated in Maranhão and Alagoas. The state of Rio Grande do Norte composed the greatest reduction in fertility levels in the region, from 8.4 to 1.99, and Maranhão was characterized as the state with the highest fertility rate at the end of the period [82].

In addition to the risk factors related to changes in reproductive behaviors, it is important to highlight the factors related to lifestyle—physical inactivity, alcohol consumption, and high-calorie foods—observed in the states of this Brazilian region [5, 83–85]. The National Health Survey (*Pesquisa Nacional de Saúde*—PNS) carried out in 2013 showed the high prevalence of consumption of unhealthy foods in the Brazilian population. Although the North and Northeast regions have the lowest consumption percentages in comparison to the other regions, higher consumption of soft drinks, sweets and alcoholic beverages was observed in Pernambuco and Ceará, similar to the states in more developed regions [83]. Trends in the adoption of unhealthy eating habits have also been seen in rural areas in the Northeast region [84].

The reduction in the risk of breast cancer as result of adopting habits considered healthy has been confirmed in several meta-analyses and systematic reviews [85–88]. In the 2013 PNS, it was also found that practically half of the Brazilian population did not reach the recommendations of at least 150 minutes of physical activity a week, with a higher prevalence among women (51.5%). Regarding the country’s macro-regions, there was little variation in the practice of physical activity [89]. The industrialization and urbanization processes, which have occurred in the country since the 20th century, modified the population’s lifestyles, promoting the practice of inadequate dietary patterns and predominant sedentary occupation models [89, 90].

If there are no major changes in the PNAO, the risk of death is expected to increase during the coming periods in the younger cohorts from the states in the Northeast region. It is noteworthy that the PNAO was instituted with the objective of contemplating the actions of promotion, prevention, early detection, adequate treatment, and palliative care of cancer, in addition to guaranteeing comprehensive care to the population affected by the disease [63, 64]. However, the large centers in the country still have a large part of the offer of services for the treatment of breast cancer and there are strong indications of a shortage of the offer of care in most of the country, such as the North and Northeast regions, for example. Women who do not reside in large cities have difficulty in commuting, thus, treatments are frequently not completely implemented [13].

In addition, in 2020 and 2021 Brazil has high incidence and mortality rates due to covid-19, which impacted the treatment and control of other morbidities, including breast cancer. Women in fear of the SARS-COV-2 infection have postponed medical appointments and tests for the early detection of breast cancer. In addition, the Oncology Care Network has postponed many surgeries, with a view to reserving beds for the treatment of covid-19. We believe that this situation may have a period effect, increasing the incidence of women diagnosed with an advanced stage of the disease and thereby increasing the risk and death from breast cancer in the next five-year period (2020–2024).

Inequalities in the quality of information and coverage of death records between states in the Northeast region represent a limitation of the present study; however, corrections were made, improving the quality of the estimates that were generated. Another limitation is the problem of identifying the effects of the APC models, which are already widespread among researchers and with widely evaluated resolutions. Due to the linearity between the effects of age, period, and cohort, the adjustment allows for obtaining of infinite solutions for the models of maximum likelihood, with different estimates for its parameters, providing the same forecast for any combination of the effects, but making it impossible to estimate the complete model [16–18]. However, there is a consensus in the literature that, when using classic models, the most appropriate method for correcting the problem is that of estimable functions, as used in the present study [16–18]. Furthermore, it was not possible to explicitly assess the effect of risk and protective factors in the models coincidingly correlated with breast cancer incidence and mortality, namely: fertility rate; prevalence of breastfeeding; prevalence of oral contraceptive use; prevalence of inadequate food consumption; prevalence of smoking; and prevalence of alcohol consumption, since this information is not available for the combination of age and period. However, based on the literature, it is possible to identify changes in these risk and protective factors over time, and thus it is possible to raise hypotheses about which factors may be correlated with the temporal trend of disease mortality and the temporal effects of age, period, and cohort.

The main limitation of ecological studies is the impossibility of evaluating the association between the outcome and risk factors, as they present aggregated data on the outcome and exposure, and thus, it is not possible to extrapolate their findings to the individual level (ecological fallacy). However, it allows us to raise hypotheses about possible contextual factors that may be correlated with the outcome, and these hypotheses can be tested in observational studies with individual data about the outcome and associated factors [15–17].

## Conclusion

The highest rates of mortality from breast cancer in all the five-year periods studied were observed in the locations that had the best socioeconomic conditions and access to health services, with a progressive increase in the coefficients of mortality and risk of death in the five-year period of the 2000s, in women born after the 1950 cohort. Such findings may be correlated with the increased prevalence at the population level of risk factors for breast cancer, which was not accompanied by public health measures to promote early detection and timely treatment of the disease, contributing to the increase in mortality rates by this neoplasm over time, especially in women from younger cohorts.

## Supporting information

S1 Fig Breast cancer mortality rates by age group and death cohort in Northeast state Brazil (Alagoas, Bahia), 1980–2019. (TIF)

S2 Fig Breast cancer mortality rates by age group and death cohort in Northeast state Brazil (Ceará, Maranhão), 1980–2019. (TIF)

S3 Fig Breast cancer mortality rates by age group and death cohort in Northeast state Brazil (Paraíba, Pernambuco), 1980–2019. (TIF)

S4 Fig Breast cancer mortality rates by age group and death cohort in Northeast state Brazil (Piauí, Rio Grande do Norte), 1980–2019. (TIF)

S5 Fig Breast cancer mortality rates by age group and death cohort in Northeast state Brazil (Sergipe), 1980–2019. (TIF)
