# Supplementary figures and images for: Mortality due to breast cancer in a region of high socioeconomic vulnerability in Brazil: Analysis of the effect of age-period and cohort
Source: PLoS One. 2021 Aug 13;16(8):e0255935. doi: 10.1371/journal.pone.0255935 (PMC8362978; doi:10.1371/journal.pone.0255935)

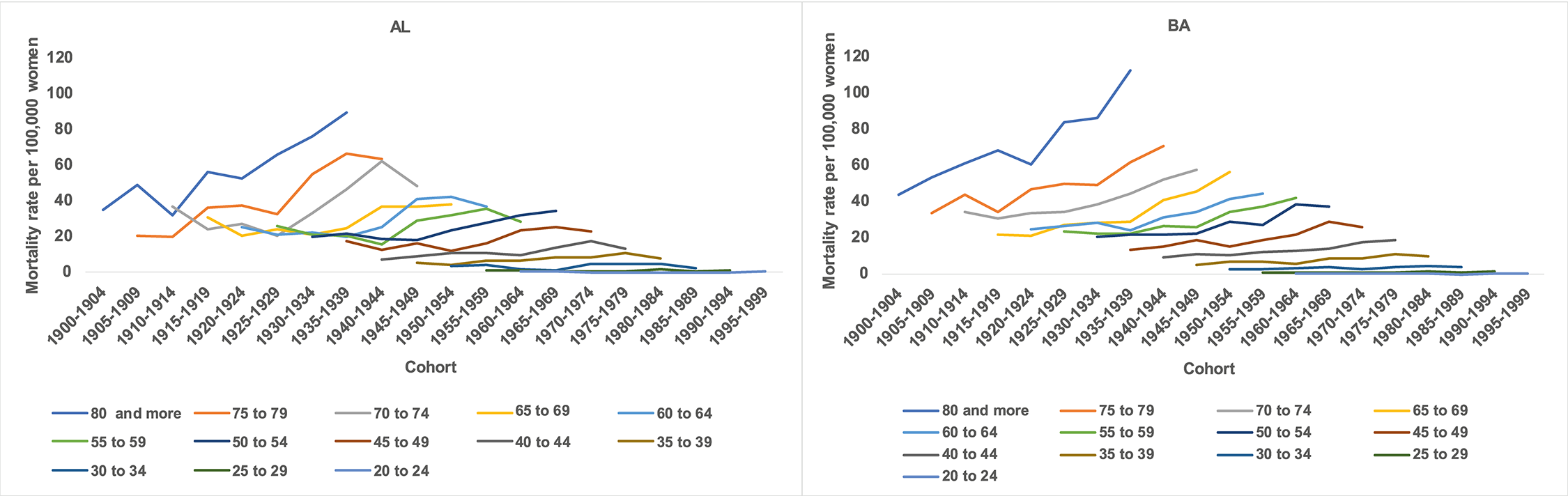

Supplement: S1 Fig — (TIF) [file pone.0255935.s001.tif]

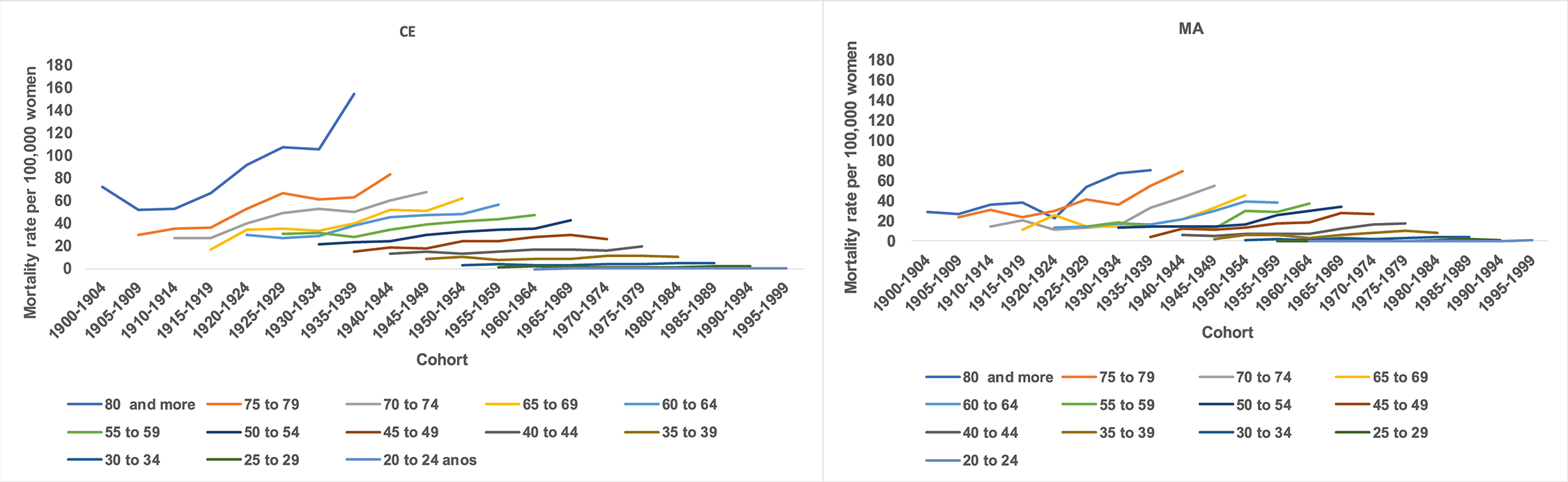

Supplement: S2 Fig — (TIF) [file pone.0255935.s002.tif]

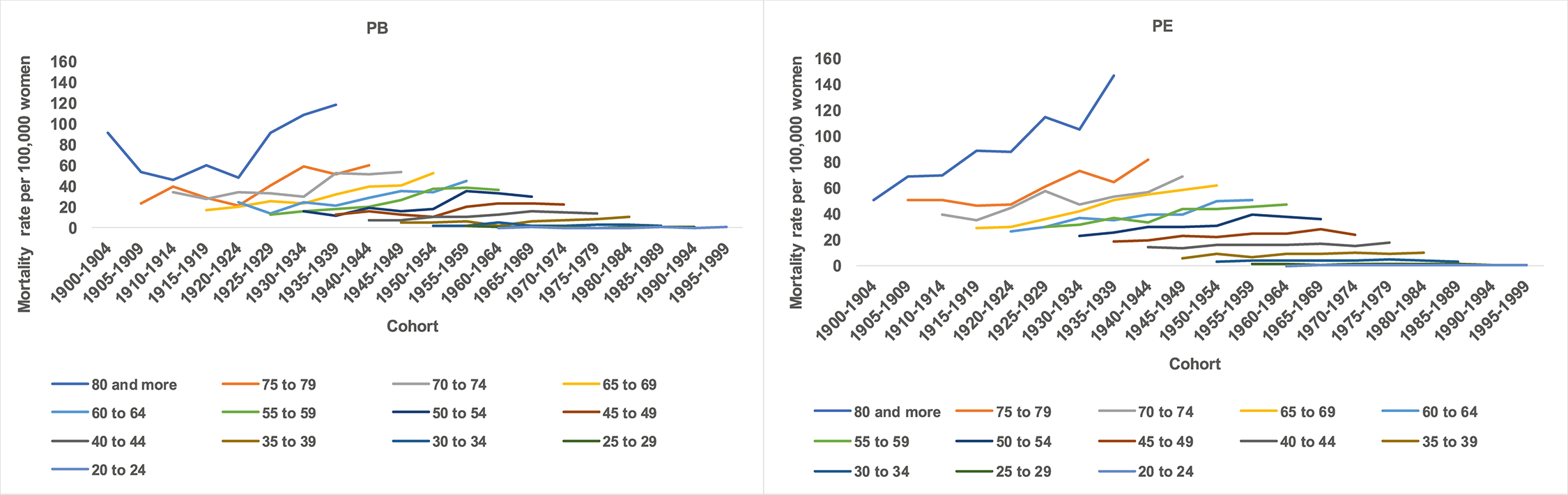

Supplement: S3 Fig — (TIF) [file pone.0255935.s003.tif]

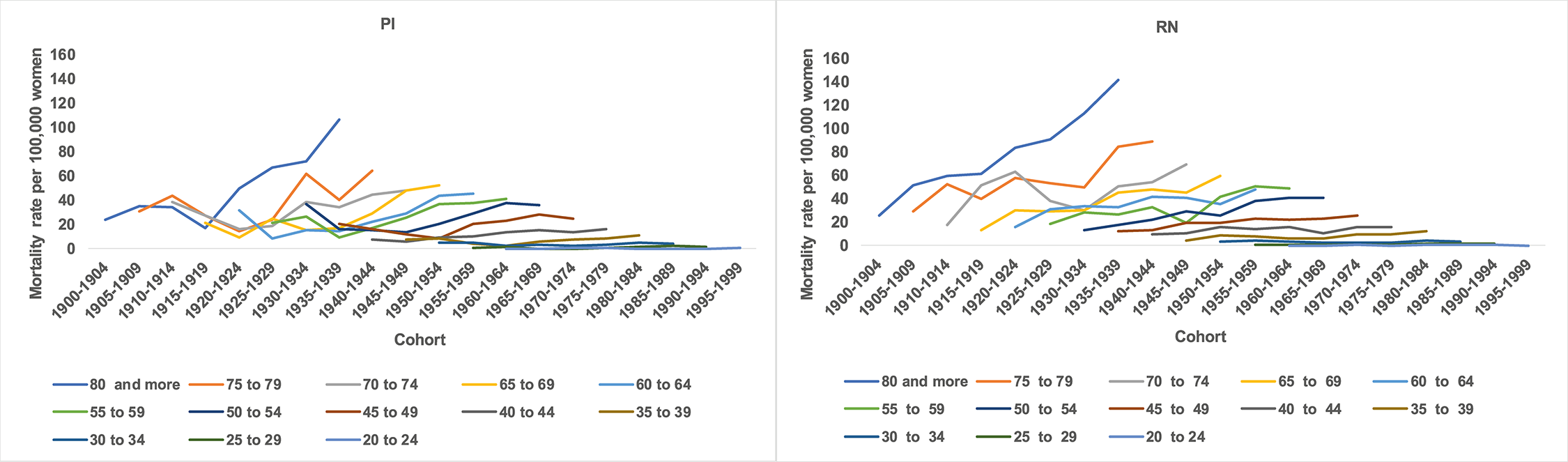

Supplement: S4 Fig — (TIF) [file pone.0255935.s004.tif]

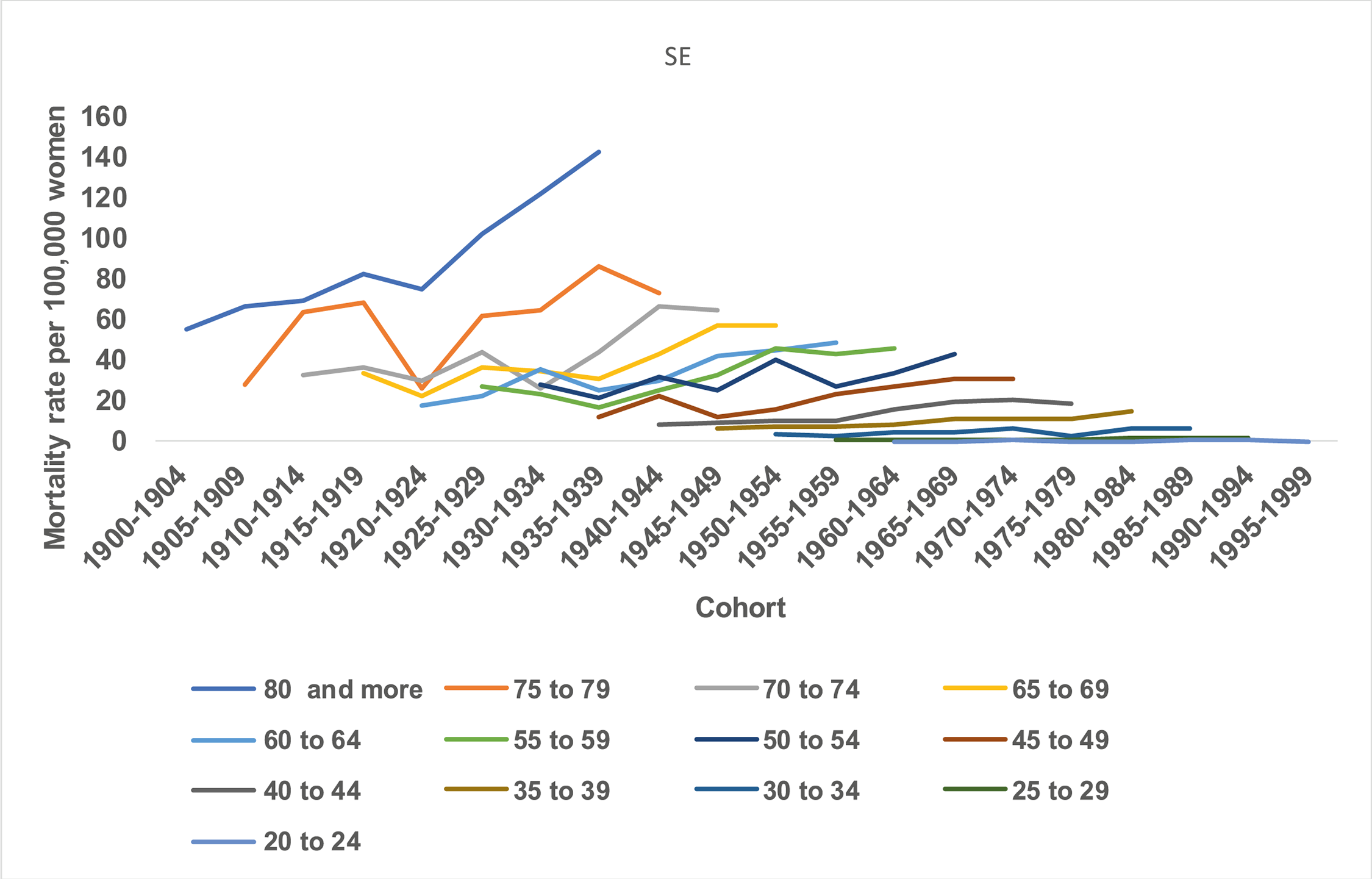

Supplement: S5 Fig — (TIF) [file pone.0255935.s005.tif]
